# High levels of mannose-binding lectin are associated with lower pulse wave velocity in uraemic patients

**DOI:** 10.1186/1471-2369-15-162

**Published:** 2014-10-04

**Authors:** Mads Hornum, Jakob T Bay, Peter Clausen, Jesper Melchior Hansen, Elisabeth R Mathiesen, Bo Feldt-Rasmussen, Peter Garred

**Affiliations:** Department of Nephrology, Copenhagen University Hospital, Rigshospitalet, Copenhagen, Denmark; Laboratory of Molecular Medicine, Department of Clinical Immunology Sect. 7631, Copenhagen University Hospital, Rigshospitalet, Copenhagen, Denmark; Department of Nephrology, Herlev Hospital, Copenhagen University, Herlev, Denmark; Department of Endocrinology, Copenhagen University Hospital, Rigshospitalet and University of Copenhagen, Copenhagen, Denmark

**Keywords:** Arterial stiffness, Augmentation index, Endothelial function, Glucose intolerance, Mannose-binding lectin

## Abstract

**Background:**

Uraemia is associated with a highly increased risk of cardiovascular disease. Mannose-binding lectin (MBL) has been shown to be involved in cardiovascular pathophysiology and a protective effect of MBL is suggested. The purpose of the present study was to evaluate a potential impact of MBL on vascular parameters in uraemic patients.

**Methods:**

A cohort of 98 patients with end stage renal disease (ESRD) awaiting kidney transplantation had pulse wave velocity (PWV) and augmentation index (AIX) examined by tonometry and endothelial dependent flow-mediated (FMD) and endothelial independent nitroglycerin-induced (NID) dilatory capacities of the brachial artery measured by ultrasound. An oral glucose tolerance test (OGTT) was performed and serum levels of MBL were measured using Luminex xMAP bead array technology.

**Results:**

The cohort was divided in two groups according to MBL-concentration below or above the median concentration. These groups were comparable regarding age, BMI, and duration of ESRD. PWV was significantly lower in the group with high MBL levels compared to the group with low MBL levels and trends toward better AIX and higher insulin sensitivity (ISI) was also seen in the group with high MBL levels. No difference was seen in FMD and NID.

**Conclusions:**

High levels of MBL are associated with lower PWV and the use of antihypertensive drugs in a cohort of patients with ESRD awaiting kidney transplantation suggesting a beneficial role of high levels of MBL on arterial stiffness in uraemia.

## Background

Uraemia is associated with a highly increased risk of cardiovascular disease. Vascular function in uraemic patients is severely damaged by a number of factors including increased phosphate, hypertension and increased calcification of large arteries. Stiffness of elastic large arteries as measured by pulse wave velocity are associated with increased mortality [1, 2]. Hypertension and age seems to be especially important for the development of arterial stiffness in non-diabetic uraemic patients [3]. Other factors like inflammation, infections and hyperlipidaemia damage the endothelial function as measured by flow mediated vasodilatation in the brachial artery [4, 5]. Genetically determined variation in MBL- serum concentration has been shown to affect the clinical course of cardiovascular disease, and an impairment of endothelial function caused by low MBL concentrations has been described in childhood infection [6]. Potential effects of MBL concentrations on arterial stiffness are not well described and especially the potential influence of MBL in the deterioration of arterial and endothelial function in uraemia needs further investigation.

The aim of the present study was to evaluate a potential impact of MBL on vascular parameters in uraemic patients.

## Methods

This is a prospective, observational cohort study including 98 patients with a scheduled living donor kidney transplantation (n = 48) or deceased donor transplantation on the waiting list for transplantation (n = 50) in the period between January 2006 and March 2008 at the two transplantation centres in Copenhagen (Copenhagen University Hospital, Rigshospitalet and Herlev Hospital (Tx-group)). To be included the patients had to be between 18 to 65 years of age with no more than one previous kidney transplantation.

The Scientific-Ethical Committee of the Capital Region of Denmark (# KF 01279825) and The Data Protection Agency (#2006-41-5640) approved the study. All participants gave their informed written consent.

### Study procedure

All participants were examined before kidney transplantation. The majority of the patients were anuric and the few with remaining diuresis all had an estimated baseline creatinine clearance below 15 ml/min. The examinations were performed after an overnight fast including coffee, tobacco, and exercise abstinence for 10 hours. Antihypertensive medication was allowed in the morning. The examiners were blinded regarding the actual clinical and metabolic status of the patient at the time of examination and all clinical information was analyzed and described after data collection was completed. Data regarding duration of smoking history, ESRD, antihypertensive treatment were obtained by interview and from the medical record. Weight and height were measured.

Hemodialysis patients were examined between days of hemodialysis. Peritoneal dialysis patients had the peritoneal fluid (PF) drained in the morning at 6 a.m. The OGTT was performed more than 12 hours after last instillation of PF and more than 4 hours after draining the PF. After 10 minutes in the supine resting position blood pressure was measured in the arm opposite to a fistula or dialysis catheter. Mean arterial blood pressure (MAP) was given as mean of three measurements. Fasting blood samples were drawn in an antecubital vein from the same arm for the determination of plasma (p)-glucose (PG), p-CRP and p-homocystein. PG concentrations were analysed by the glucose-hexokinase method (Gluco-quant®, Roche Diagnostics GmbH, D-68298 Mannheim, Germany) and p-insulin were measured using enzyme-linked immunosorbent assay kits (Elecsys, Roche Diagnostics GmbH, D-68298 Mannheim, Germany). All assays were automated and performed on a Cobas Fara robot (Roche Diagnostics GmbH, Mannheim, Germany).

### Measurement of serum MBL

Serum MBL concentrations were measured using an anti-MBL monoclonal antibody double sandwich technique based on the Luminex xMAP bead array technology as previously described [7]. In healthy subjects the median day-to-day variability in MBL concentrations expressed as CV was 6%. The lower detection level was 5 μg/L and intra- and interassay CVs were below 5 and 10%, respectively.

### MBL2 genotypes

Blood collected in 10 ml EDTA containing vacutainers was used for DNA extraction. Anticoagulated blood was frozen at -80°C before DNA processing. MBL2 single nucleotide polymorphisms (SNPs) in form of the structural variants named B (codon 54, rs1800450, C (codon 57, rs1800451, and D (codon 52, rs5030737) were typed by PCR using sequence specific priming (PCR-SSP) as previously described [8]. The normal allele is designated "*A*". All three structural variant alleles (*B*, *C*, and *D*) have a considerable effect on MBL concentrations and to avoid small groups, the three alleles were grouped in one category called allele "*O*" for statistical analyses.

Standard laboratory methods were applied for the analysis of the other samples.

### Pulse wave analysis and velocity

PWV and AIX of the carotid-femoral pulse wave was recorded sequentially at the carotid and femoral artery using applanation tonometry (SphygmoCor®; AtCor Medical, Sydney, NSW, Australia) performed by the same observer (MH) as previously described in detail [3]. The reproducibility of AIX and PWV for two repeat scans on two different days in ten uraemic patients from the present study was analysed using the Bland Altman method. The mean ± SD intra-observer difference was 0.1 ± 0.7 m/s for PWV, 1.7 ± 5.5 % for AIX, which is in accordance with other groups [4, 9].

### Flow mediated vasodilatation (FMD) and nitroglycerin-induced dilatation (NID)

An Acuson 128XP/10™ ultrasound system with extended frequency-imaging option and a 7-MHz linear transducer was used to measure the brachial artery dilatory responses to increased flow (FMD) and to nitroglycerin (NID) based on previous published methodology by our group and others [4, 9, 10].

External ultrasound measurements of FMD and NID have previously been shown to provide accurate and reproducible results. Coefficients of variation of the diameter changes expressed as diameter in percentage of the baseline scan diameter (100%) in the present study was 4.4 and 4.1% for FMD and NID for eight scans of the same subject from the present study population (within patient variability) performed on separate days within a period of 2 weeks by the same investigator, similar to previous reports [9, 11].

### Evaluation of glucose tolerance

A 75-gram OGTT was performed according to the WHO criteria [12] and an insulin sensitivity index (ISI) was calculated as previously described in details [13].

### Antihypertensive treatment

Antihypertensive treatment mainly included beta-blockade, calcium channel blockade, angiotensin-II-blockade and diuretics.

### Statistics

Data analyses were performed using Statistical Analysis Software (SAS®) version 9.1. Unless specified otherwise, continuous data is described as mean ± standard deviation (SD) or median and range for normal and skewed distributions, respectively. The Shapiro-Wilk W test and a normal plot were used to judge normality. Group comparisons of continuous data were performed using unpaired t-test test for normally distributed data or non-parametric Wilcoxon Rank Sum Test or Kruskal-Wallis non-parametric one way analysis of variance test. Chi square or Fisher’s exact tests were used for group comparisons between categorical data where appropriate. A p value of <0.05 was used to determine significance.

## Results

The cohort was divided in two groups according to MBL concentration below or above the median concentration. These groups were comparable regarding age, hypertension, glycemic status, BMI, and duration of ESRD.

Univariate analysis showed no association between the MBL level and PWV, ISI, FMD, NID or augmentation index in the whole group but the variance analysis between the two groups demonstrated that PWV was significantly lower in the group with high MBL levels compared to the group with low MBL levels (Table 1, p = 0.03) and trends toward better AIX and higher ISI was also seen in the group with high MBL levels. No difference was seen in FMD or NID. Systolic and diastolic blood pressure was similar between groups, however the use of antihypertensive drugs were significantly higher in the group with the lowest PWV and highest MBL (Table 1).

**Table 1:** **Comparison of clinical and demographic data between patients with a MBL level above or below the median**

	MBL < 722 ug/L	MBL >722 ug/L
	N = 49	N = 49
Age (years)	45.3 ± 13.0	41.6 ± 12.4
ESRD (months)	29 (0–168)	33 (0–120)
Dialysis status:		
Haemodialysis (N)	34	32
Peritoneal dialysis (N)	13	13
Pre-dialysis (N)	2	4
Haemodialysis, venous access: Arterio-venous fistulae/permanently haemodialysis catheter (N)	15/19	15/17
Tx status (living donor/deceased donor)	22/27	26/23
Myocardial infarction (N)	2	2
Smoking (N)	15	14
Stroke (N)	3	2
Diabetes (N)	3	5
Impaired fasting glucose (N)	1	2
Impaired glucose tolerance (N)	21	13
Normal glucose tolerance (N)	24	29
Fasting plasma glucose (mmol/L)	5.0 ± 0.4	5.1 ± 0.5
OGTT 120min(mmol/L)	8.0 ± 2.1	7.7 ± 2.1
ISI (index)	7.4 ± 4.1	7.9 ± 4.9
BMI (kg/m^2^)	24.2 ± 3.8	24.5 ± 3.6
PWV (m/s)	8.2 (5.6-15.8)	6.8 (5.2-16.4)*
AIX (%)	27 (-10;56)	20 (-7;50)
FMD (%)	3.8 (-8.3;19.2)	3.5 (-6.8;19.1)
NID (%)	10.5 (2.9;27.1)	11.5 (2.0;27.2)
Systolic blood pressure (mmHg)	143 ± 25	140 ± 19
Diastolic blood pressure (mmHg)	85 ± 12	84 ± 13
Number of antihypertensive drugs	2 (0–5)	3 (0–6)*
P-C-reactive protein (mg/L)	2 (1–40)	2 (1–21)
P-Homocystein (umol/L)	21 (11–62)	22 (12–150)
P-PTH (pmol/L)	21 (2–100)	20 (4–156)
P-ionized calcium (mmol/L)	1.19 ± 0.09	1.21 ± 0.09
P-phosphate (mmol/L)	1.8 ± 0.4	2.0 ± 0.6

Data are presented as mean (SD) or median and range.

MBL: Mannose Binding Lectin, ESRD: end-stage renal disease,

Tx: transplantation, BMI: body mass index, PTH: parathyroidea hormone,

OGTT: oral glucose tolerance index, ISI: insulin sensitivity index.

PWV: pulse wave velocity,

AIX: Augmentation Index, FMD: flow mediated vasodilatation,

NID: nitroglycerin independent dilatation,

*P < 0.05.

Data on myocardial infarction, stroke and smoking were collected and there was no difference between the groups (Table 1).

The variance analysis was also performed for the whole group with factors known to influence PWV, describing 65% of the variance and as expected age, hypertension, glucose intolerance, insulin resistance and homocystein was significantly associated with PWV, but MBL levels just did not reach statistical significance in this analysis (Table 2).

**Table 2:** **Results of an ANOVA of factors obtained at baseline in 98 ESRD patients awaiting kidney transplantation the model describes 65% of the variance in PWV (** ***P < 0.0001*** **)**

Parameter	Effect on PWV(m/s)	95% confidence intervals	***P***-value
Intercept	-0.14	-3.66; 3.38	0.94
Age (years)	0.12	0.08; 0.16	<0.0001
BP >130/80 mmHg	1.76	0.81; 2.72	0.0005
BP < 130/80 mmHg (reference group)	0		
Number of antihypertensives (n)	-0.08	-0.45;0.29	0.65
ISI (index)	0.21	0.07; 0.36	0.0043
OGTT120min	0.33	0.09; 0.56	0.0067
Homocystein (umol/L)	0.03	0.01; 0.05	0.01
MBL Genotype A/A	1.23	-1.21; 3.66	0.32
MBL Genotype A/O	0.18	-1.85; 2.21	0.86
MBL Genotype O/O (reference group)	0		
LogMBL	-0.88	-1.85; 0.10	0.0764
Female sex	0.31	-0.81; 1.42	0.58
Male sex (reference group)	0		
ESRD (months)	-0.01	-0.03; 0.01	0.22
Augmentation Index	-0.01	-0.05; 0.03	0.51
CRP (mg/L)	-0.04	-0.11; 0.04	0.32

Kruskal-Wallis one way of variance analysis of the effect on pulse wave velocity (PWV) using factors obtained at baseline. BP: blood pressure; ISI: insulin sensitivity index; OGTT: oral glucose tolerance test; MBL: mannose-binding lectin; ESRD: end-stage renal disease; CRP: C-reactive protein.

**Figure 1:**
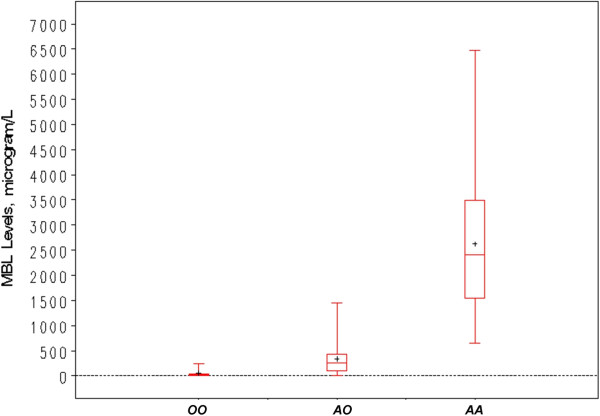
**MBL levels according to MBL2 genetic variants.** The figure illustrates median, interquartile range and minimum/maximum MBL levels stratified by OO/AO/AA MBL2 genetic variants.

Distribution of the genetic MBL2-variants differed as result of the stratification according to MBL below or above the median. The normal *A/A* genotype dominated in the group above the median (44 vs. 5), whereas patients heterozygous for MBL2 variants (*A/O*) haplotype were predominant in the group below the median (41 vs. 5). Those heterozygous for the structural variants, the O/O genotype, were found only in the group below the median (6 vs. 0), (Table 3). The difference in MBL concentrations between the MBL2 variants is shown in Figure 1.

**Table 3:** **Genetic MBL2-variants stratified according to MBL levels**

MBL2-variant	MBL < 722 ug/L	MBL > 722 ug/L
A/A	2	44
A/O	41	5
O/O	6	0

The distribution of haemodialysis or peritoneal dialysis, and the use of an arterio-venous fistulae or a permanently haemodialysis catheter, was determined in the cohort, and there were no statistical difference in PWV, ISI, FMD, NID or augmentation index between the groups divided by MBL levels.

## Discussion

In a cohort of patients with ESRD awaiting kidney transplantation we found that high levels of MBL and increased use of antihypertensive drugs were associated with lower PWV. There may be a beneficial role of high levels of MBL on arterial stiffness in uraemia which was independent of BMI, length of uraemia and age. Previously Cheung et al. [14] demonstrated that the MBL genotype influenced arterial stiffness in Chinese children with Kawasaki Disease. The children with low level MBL expression IL/LL-genotypes without coronary complications had faster PWV/stiffer peripheral conduit arteries than children with high level MBL expression HL- genotypes indicating that a high level of MBL had a protective effect on arterial function. In a study of healthy Danish blood donors aged 18–65 years, the median value of MBL was 967.5 ug/L (8–4693 ug/L) [7]. Arterial stiffness estimated by PWV [15] reflects damage of the elastic tissue of aorta [15, 16] and is associated with increased mortality in ESRD-patients [17, 18]. In a cohort of 66 uraemic patients we previously reported that age and mean arterial pressure but not pre-diabetes were independently associated with increased PWV [3] and an age and BMI matched healthy control group previously studied by our group had significant lower blood pressure and a better augmentation index, FMD and NID compared to uraemic patients on the waiting list for Tx [19]. In the present cohort divided by the median level of MBL there was no difference in age, fasting glucose, 2-hour blood glucose, ISI or blood pressure between the groups, however more antihypertensive drugs were used in the group with high MBL levels and the lowest PWV, indicating that the role of MBL in uraemia seems independently of traditional risk factors for an increased PWV. Although not significant, both augmentation index and ISI index was more favourable in the high MBL group, also suggesting a better arterial function in this group, and a more powerful study including more patients might confirm this association.

The duration of ESRD in the groups divided by the median MBL was not significantly different, an important point, since duration of haemodialysis increases MBL levels as demonstrated by Satomura et al. [20].

Apart from influencing arterial stiffness, MBL deficiency is also known to be associated with severe atherosclerosis [21] and doubles the risk for myocardial infarction (MI) in young relatively healthy Caucasians [22], and leads to cardiovascular events in type 2 diabetic South Asians [23]. Furthermore, MBL deficiency has been shown to be unfavourable in type 2 diabetic Caucasians with MI [24]. Although these studies all were performed on patients with normal renal function, they demonstrated that especially the combination of genotype O/O and impaired glucose metabolism was a hazardous combination with impact on cardiovascular events. In haemodialysis patients low MBL levels has been found to be associated with increased all-cause mortality [25]. Recently it was shown that both low MBL levels and low expressing MBL2 genotypes were associated with graft loss after kidney transplantation in patients without prior detectable HLA immunization [26]. This and other observations support the notion that MBL may be important to maintain cardiovascular homeostasis, particularly during chronic inflammation, including the importance of lectins in the pathogenesis of several inflammatory diseases [27]. Endothelial function has previously been found to be impaired in both uraemia [9] and in diabetes [28] and may reflect presence of vascular disease or early vascular damage, but in the present study we found no difference in FMD or NID between the two groups. Our cohorts had no signs of infection and only few diabetics in each group, partly explaining the missing difference between groups in FMD and NID. The overall tendency to infection is however increased in uraemic patients, and aggravated by MBL deficiency, with a high burden of dialysis-related infections and risk of bacteraemia during the dialysis sessions potentially leading to damage of the elastic tissue of the arteries and to an increased arterial stiffness thereby increasing the risk of arteriosclerotic vascular disease.

The MBL genotype influences the level of MBL and the AA expression genotype with the highest level of MBL would in theory have the best protection against infections, and thereby the lowest PWV and the highest FMD. This is in accordance with our findings with regard to PWV whereas no effect of MBL on FMD was demonstrated. However, PWV and FMD are not necessarily part in a unified pathophysiological vascular process in uraemia and may be differently influenced by different cardiovascular risk factors such as MBL.

The MBL-genotype A/A clearly was most represented in the high MBL-group in our cohort, potentially indicating a genetic element in the susceptibility to arterial damage in uraemic patients, although the relative few patients with the OO expression genotype limited the statistical power to detect difference between the groups.

## Conclusions

We demonstrated that high levels of MBL are associated with lower PWV and an increased use of antihypertensive drugs in a cohort of patients with ESRD awaiting kidney transplantation suggesting a beneficial role of high levels of MBL on arterial stiffness in uraemia. We hypothesize that this association will result in a decreased cardiovascular and infection related mortality in patients with high baseline MBL levels and a follow up study is planned to address this hypothesis. The strengths of the study were a carefully examined and characterized cohort, with solid glucose and arterial measurements. Estimation of insulin resistance and validated MBL measurements and determination of genetic MBL2 variants and statistical analysis completed the study. Limitations were the cross sectional design, relatively few patients and lack of follow up data.
